# Case Report: Giant coronary artery aneurysm with cardiac compression successfully managed by surgical resection and bypass in a normolipidemic patient

**DOI:** 10.3389/fcvm.2026.1839907

**Published:** 2026-06-10

**Authors:** Zhi Wen, Yong Luo, Mingwu Tian, Yan Du, Changxue Wu

**Affiliations:** 1 Department of Cardiovascular Surgery, Deyang People's Hospital, Deyang, China; 2 Department of Cardiovascular Surgery, Chengdu Third People's Hospital Chengdu, China; 3 Department of Cardiology, Deyang People's Hospital, Deyang, China

**Keywords:** case report, coronary artery bypass grafting, giant coronary artery aneurysm, multimodal imaging, right coronary artery

## Abstract

Here, we report a case of a 53-year-old woman without traditional cardiovascular risk factors who presented with acute chest pain and signs of right heart compression and superior vena cava syndrome. Multimodal imaging, including computed tomography angiography, cardiac magnetic resonance, and echocardiography, revealed a giant right coronary artery aneurysm with a maximum diameter of approximately 15.6 cm, containing a large amount of intraluminal thrombus and causing compression of the right atrium, right ventricle, and superior vena cava. Serological and pathological examinations ruled out vasculitic diseases, while histopathology of the aneurysmal wall revealed fibrous hyperplasia, architectural disorganization, and hyalinization, suggesting non-atherosclerotic, non-inflammatory arterial wall degeneration with an unclear origin. The patient underwent successful aneurysm resection, thrombus evacuation, proximal and distal ligation of the right coronary artery, and concurrent distal bypass grafting using a saphenous vein graft. Postoperatively, the central venous pressure decreased significantly, the clinical symptoms resolved rapidly, and the graft remained patent at the 3-month follow-up.

## Introduction

Giant coronary artery aneurysms (CGAAs) are rare, with an estimated incidence of <0.02% (1). Although there are no universally accepted criteria, a coronary artery diameter greater than 2 cm in adults is commonly considered “giant.” Alternative definitions describe an aneurysm as being more than four to five times the normal vessel diameter or having a maximum dimension exceeding 8 cm (2). Atherosclerosis is the most common cause, followed by congenital anomalies, Kawasaki disease, and vasculitides (3). GCAAs may be asymptomatic or present with complications, including thrombosis, embolism, rupture, or fistula (4). Compression of adjacent cardiac chambers is an uncommon but hemodynamically significant finding.

Our patient, with a maximum diameter of 15.6 cm, clearly met all proposed definitions of a giant coronary artery aneurysm, presenting with acute chest pain and obvious signs of superior vena cava syndrome due to compression of the cardiac chambers. We report this case as follows, with the cited data and de-identified images obtained through the patient's written consent.

## Case description

A 53-year-old woman with no significant medical history was admitted with acute retrosternal chest pain lasting 24 h. The pain was paroxysmal, with each episode lasting approximately 30 min, radiating to both shoulders and the upper back, and was accompanied by profuse diaphoresis. She had no history of Kawasaki disease, chest trauma, or cardiac symptoms. On presentation, her vital signs were as follows: blood pressure = 118/76 mmHg; heart rate = 92 beats per minute (regular); respiratory rate = 18 breaths per minute; oxygen saturation = 96% on room air; and tympanic temperature = 36.7 °C. Among the notable findings were distended external jugular veins when in the supine position, along with prominent, non-pulsatile subcutaneous venous collaterals over the anterior chest and abdominal walls (Supplementary Figure S1A). Cardiac auscultation revealed distant heart sounds without murmurs, rubs, or gallops. No hepatomegaly or peripheral edema was observed. The Pemberton sign (facial congestion and dyspnea upon arm elevation) was not observed. The electrocardiogram (ECG) showed a normal sinus rhythm at 92 beats per minute, with no ST-segment elevations or depressions but it revealed pathological Q waves in leads III and aVF. Although the initial ECG did not indicate acute ischemia or prior myocardial infarction, the later emergence of Q waves suggested a possible prior silent infarction in the inferior territory. Initial laboratory studies showed a significantly elevated high-sensitivity cardiac troponin I level of 18.216 ng/mL (institutional upper reference limit: 0.039 ng/mL for women). Her fasting lipid profile revealed total cholesterol of 154.7 mg/dL, low-density lipoprotein (LDL) cholesterol of 93.6 mg/dL, high-density lipoprotein (HDL) cholesterol of 44.1 mg/dL, triglycerides of 83.3 mg/dL, and apolipoprotein A-I of 113 mg/dL (normal range: 76–214 mg/dL). The HbA1c level was 5.2% (normal <5.7%). The complete blood count showed a normal white blood cell count (6.8 × 10^9^/L), hemoglobin level (13.2 g/dL), and platelet count (256 × 10^9^ /L). The chemistry panel revealed normal renal function (creatinine = 0.71 mg/dL and blood urea nitrogen = 12 mg/dL), liver function tests within normal limits, and a C-reactive protein level of 0.3 mg/L (normal <5 mg/L). Arterial blood gas analysis on room air showed the following: pH = 7.41; PaCO_2_ = 38 mmHg; PaO_2_ = 94 mmHg; ${\mathrm{HCO}}_{3}^{-}$ = 24 mmol/L; and lactate level = 1.2 mmol/L. Infectious etiologies were ruled out by negative serologies for syphilis [rapid plasma reagin (RPR) and Treponema pallidum hemagglutination assay (TPHA)], negative Quantiferon-TB Gold test, and negative PCR for SARS-CoV-2, and cytomegalovirus.

Upon arrival at the emergency department, the patient underwent an immediate clinical evaluation. Given the acute chest pain accompanied by diaphoresis and elevated high-sensitivity cardiac troponin I, acute coronary syndrome was initially suspected. To rapidly evaluate coronary artery pathology and thoracic aortic emergencies, a bedside electrocardiogram was initially performed, followed by coronary computed tomography angiography (CTA) within 2 h as the initial advanced imaging technique. Coronary CTA revealed a massive, heterogeneously enhancing mass measuring 12.6 cm × 8.3 cm × 12.3 cm located in the anterior mediastinum, continuous with the proximal right coronary artery (RCA). The mass contained linear calcifications and a large non-enhancing central area consistent with a thrombus, causing significant compression of the right atrium, right ventricle, and superior vena cava (Figure 1). Transthoracic echocardiography revealed a heterogeneous mass measuring 135 mm × 98 mm × 77 mm, located adjacent to the right atrial free wall, with anechoic zones and focal calcification. There was significant compression of the right atrium, restricted tricuspid valve opening with mild regurgitation, and a dilated inferior vena cava with reduced collapsibility (less than 50%), indicating elevated right atrial pressure. The left ventricular function was preserved, with a left ventricular ejection fraction of 54% (Supplementary Figure S2).

**Figure 1:**
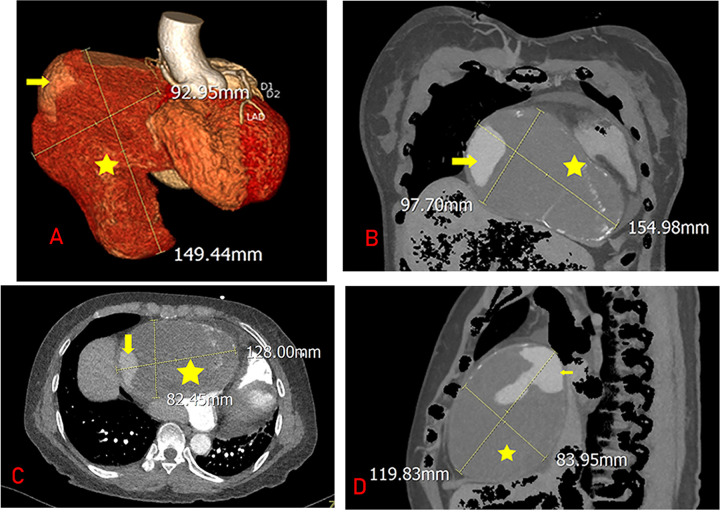
Coronary CTA showing the morphology and measurements of the aneurysm from different angles. (A) Axial CTA showing compression of the right atrium; (B) Coronal view demonstrating RCA origin; (C) 3D reconstruction with aneurysm size; (D) Volume-rendered image showing thrombus. *Yellow arrows:* blood flow within the aneurysm; *yellow five-pointed star:* intraluminal thrombus.

Cardiac magnetic resonance (CMR) further characterized the mass, measuring 15.6 cm × 10.2 cm, and revealed mixed signal intensities on T1- and T2-weighted images. Bright-blood cine imaging confirmed the flow communication with the RCA. The post-contrast sequences revealed enhancement consistent with the great vessels, confirming the aneurysmal nature and ruling out the possibility of a solid tumor (Supplementary Figure S3). Coronary angiography, the diagnostic standard, confirmed a giant aneurysm of the proximal RCA. The distal RCA was occluded but was filled retrogradely via robust collaterals from the left coronary system (Supplementary Video S1). Serological tests for autoimmune and vasculitic disorders were negative, effectively ruling out inflammatory etiologies.

The patient underwent an urgent surgical repair. After performing a median sternotomy and cardiopulmonary bypass with the heart still beating, a 15 cm × 8 cm conical aneurysm was identified. It had a thick fibrous capsule and was densely adherent to and compressed the right atrium and ventricle. The aneurysmal sac was incised, and a large volume of mixed-stage laminated thrombi was evacuated. The proximal and distal RCA ostia were ligated using 4-0 polypropylene sutures. A saphenous vein graft was anastomosed from the ascending aorta to the distal RCA to restore the perfusion. A subtotal adventitiectomy was performed, and the residual sac wall was oversewn to achieve hemostasis (Figure 2). The central venous pressure decreased from 16.7 cmH_2_O preoperatively to 4.3 cmH_2_O postoperatively (Supplementary Figure S4), with extubation achieved on postoperative day 1. The prominent chest wall venous collaterals had resolved by postoperative day 3 (Supplementary Figure S1B), and the patient was discharged on postoperative day 7 with a regimen of long-term dual antiplatelet therapy. Histopathological examination of the aneurysmal wall revealed fibrous tissue hyperplasia with architectural disorganization, focal hyalinization, myxoid degeneration, calcific deposits, hemorrhagic foci, and hemosiderin-laden macrophages (Figure 3). At the 3-month follow-up, the patient was asymptomatic. A surveillance coronary CTA confirmed that the lumen in the saphenous vein graft remained patent (Supplementary Figure S5) (see Table 1 for details).

**Figure 2:**
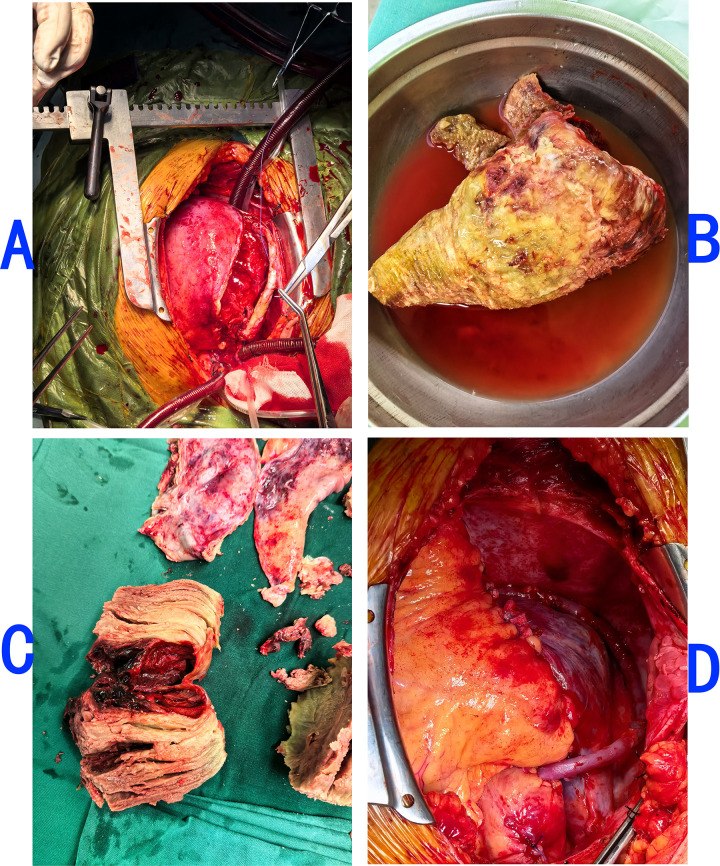
Intraoperative findings: aneurysm, thrombus, and surgical repair. **(A)** Incision of the aneurysmal wall, revealing a large, organized thrombus. **(B)** Extracted intact thrombus. **(C)** Resected aneurysm wall with dissected laminar thrombus. **(D)** Final operative view.

**Figure 3:**
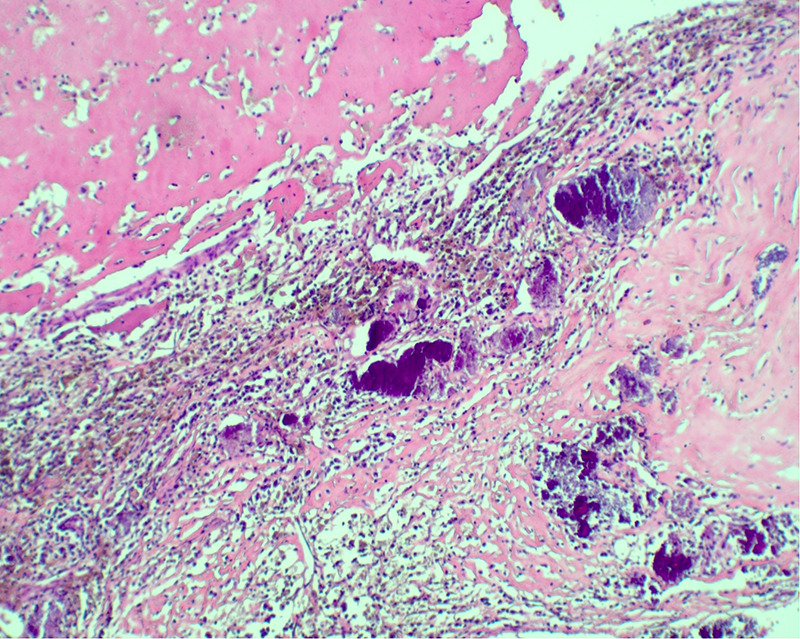
Histopathology of the aneurysmal wall (H&E staining, digital slide scanned at 20× magnification): fibrous hyperplasia with architectural disorganization, hyalinization, myxoid degeneration, calcification, hemorrhage, hemosiderin deposition, and lymphoplasmacytic infiltration, accompanied by thrombus formation.

**Table 1:** Timeline.

Timepoint	Event
Day 1	Admitted with acute retrosternal chest pain of 24 h duration. Initial laboratory tests revealed elevated high-sensitivity cardiac troponin I. Lipid profile was normal.
Day 2	Coronary CTA revealed a giant right coronary artery aneurysm compressing the right atrium, right ventricle, and superior vena cava.
Day 3	Transthoracic echocardiography and CMR were performed, confirming the aneurysmal nature and ruling out a solid tumor. Serological tests for autoimmune and vasculitic disorders were negative.
Day 4	Coronary angiography confirmed a giant aneurysm of the proximal right coronary artery with distal occlusion and robust collateral circulation in the left coronary system.
Day 8	Underwent urgent surgical repair, including aneurysm incision and thrombus evacuation, ligation of the proximal and distal RCA ostia, and saphenous vein grafting from the ascending aorta to the distal RCA. After the surgery, the patient was transferred to the intensive care unit.
Day 9	Extubated on postoperative day 1.
Day 15	Discharged home on long-term dual antiplatelet therapy.
Day 10	At the 3-month follow-up, coronary CTA showed a patent saphenous vein graft with good distal runoff.

## Discussion

Coronary angiography, historically regarded as the gold standard for defining coronary anatomy, significantly underestimated the true size of the aneurysm due to the presence of extensive intraluminal thrombus, a well-recognized limitation (5). This pitfall reinforces the necessity of a multimodal imaging approach. CTA provides precise anatomical delineation, three-dimensional reconstruction, and definitive confirmation of the thrombus. CMR offers superior soft tissue characterization, which is critical for excluding neoplastic processes by demonstrating flow communication and enhancement patterns consistent with those of the great vessels (6). Echocardiography facilitates the assessment of an aneurysm's relationship with cardiac structures and hemodynamic changes, including valvular function and systolic/diastolic function. In this case, it revealed compression in the cardiac chambers and elevated the right heart pressure. Multimodal imaging is invaluable for evaluating giant coronary artery aneurysms. CTA and CMR complement each other diagnostically: CTA provides detailed anatomy and thrombus burden, while CMR provides tissue characterization and hemodynamic information. In certain situations, surgery can be performed first without relying on invasive angiography (7). The combined use of these three modalities not only prevents misdiagnosis of giant aneurysms as cardiac tumors or other lesions, thereby reducing diagnostic errors, but also ensures sufficient diagnostic certainty for the surgical treatment of complex coronary artery diseases (8). The large size (15.6 cm) of this mediastinal mass and its mass effect leading to cardiac compression necessitated a broad differential that required multimodal imaging for resolution. Principal considerations included cardiac tumors (myxomas, sarcomas, or lymphomas), which were ruled out by CMR, demonstrating flow communication with the RCA and enhancement patterns matching the great vessels. Pericardial cysts, typically unilocular and lacking flow communication, were also included. Abnormalities in the coronary venous system, such as giant aneurysms of the coronary sinus or a persistent left superior vena cava with aneurysmal dilation, require careful delineation of the lesion's origin from the RCA on CTA and angiography to distinguish from arterial etiologies (9). Other vascular lesions included pulmonary artery aneurysm, aortic dissection with intramural hematoma, or a ruptured sinus of Valsalva aneurysm.

Etiological considerations merit a detailed examination. Atherosclerosis is the predominant cause of coronary artery aneurysms in adults (3). Approximately 20%–30% of coronary artery aneurysm (CAA) cases can be classified as congenital abnormalities, pathologically characterized by extensive destruction of elastic and collagen fibers in the aneurysmal wall, along with disruption of the elastic lamina (10, 11). However, in this patient, a normal lipid profile and the absence of angiographically significant coronary stenoses rendered a purely atherosclerotic etiology less plausible. Serological tests for systemic vasculitides, including Kawasaki disease and Takayasu arteritis, yielded negative results. Histopathological analysis of the resected aneurysmal wall revealed fibrous tissue hyperplasia, architectural disorganization, myxoid degeneration, and focal hyalinization without classic atheromatous components. These findings suggest a non-atherosclerotic, non-inflammatory, degenerative process in the arterial wall. Although some authors have proposed that giant coronary artery aneurysms may have a congenital predisposition, our histopathological findings, which show fibrous hyperplasia, architectural disorganization, and hyalinization without classic atheromatous components, are more consistent with an acquired or idiopathic degenerative process (12). The exact etiology remains unclear; however, possibilities include an unrecognized vasculopathy, post-inflammatory repair, or a forme fruste of a connective tissue disorder without systemic manifestations.

The absence of overt signs of right heart failure, such as hepatomegaly, peripheral edema, or ascites, despite significant right heart compression on imaging, warrants further investigation. Although the patient exhibited elevated jugular venous distension and prominent chest wall collaterals, suggesting increased right atrial pressure, overt right heart failure was not observed. This is likely attributable to the chronic, progressive nature of the mass effect, which allowed the gradual development of compensatory venous collateral circulation, as evidenced by subcutaneous venous collaterals over the anterior chest and abdominal walls, thereby alleviating the right heart pressure overload. The presenting symptom of chest pain, rather than dyspnea or signs of right heart failure, could be attributed to aneurysmal stretching of the adventitial nerve fibers, microembolization from the intraluminal thrombus, or relative myocardial ischemia due to a reduced coronary flow reserve. Management depends on the symptoms, size, thrombus burden, and mass effect. The options include the following: (1) medical management with anticoagulation/antiplatelet therapy and close surveillance for small or asymptomatic aneurysms; (2) percutaneous intervention (covered stents or coil embolization) for selected anatomies; and (3) surgical resection with or without concurrent bypass grafting, which is the standard for giant, symptomatic, or compressing aneurysms. Our patient required surgery due to a significant mass effect that was causing cardiac compression, an imminent rupture risk, and the presence of a large thrombus with the potential of embolization, rendering conservative and percutaneous approaches unfeasible. The chosen strategy consisted of aneurysm resection, thrombus evacuation, proximal and distal ligation of the native RCA, and concurrent distal bypass grafting using a saphenous vein graft, which is the standard of care for such cases (13, 14). This approach addresses both the life-threatening mass effect and the requirement for durable myocardial revascularization. The excellent postoperative outcome attests to the efficacy of this strategy, with immediate hemodynamic improvement characterized by a marked decrease in central venous pressure, rapid resolution of venous collaterals, and a patent graft at 3-month follow-up (15). The patient did not exhibit clinical stigmata of heritable connective tissue disorders, such as Marfan syndrome, Ehlers–Danlos syndrome, or Loeys–Dietz syndrome, such as joint hypermobility, skin hyperextensibility, or ocular abnormalities. However, genetic testing for mutations in genes, including FBN1, COL3A1, TGFBR1/2, and ACTA2, was not performed, which represents a limitation of this report.

The patient provided the following statement: “I came to the hospital with severe chest pain, not knowing that a giant aneurysm was compressing my heart. The surgery removed the aneurysm and restored blood flow, and within days my symptoms, including the bulging veins on my chest, disappeared. I feel completely recovered now and hope my case helps others with this rare condition get timely treatment.”

## Conclusion

This case illustrates that for patients with giant coronary artery aneurysms presenting with cardiac compression, surgical resection with concurrent coronary artery bypass grafting is a safe and effective treatment approach. Multimodal imaging, including CTA, CMR, and echocardiography, is essential for accurate diagnosis, surgical planning, and exclusion of alternative pathologies. Even in the absence of traditional risk factors or systemic vasculitis, histopathologically confirmed arterial wall degeneration may underlie the development of such giant aneurysms.

## Data Availability

The original contributions presented in this study are included in the article/Supplementary Material. Further inquiries can be directed to the corresponding author.
