# Supplementary material for: Case Report: Giant coronary artery aneurysm with cardiac compression successfully managed by surgical resection and bypass in a normolipidemic patient
Source: Front Cardiovasc Med. 2026 Jun 10;13:1839907. doi: 10.3389/fcvm.2026.1839907 (PMC13290533; doi:10.3389/fcvm.2026.1839907)
Supplement: Supplementary file 1 [file Datasheet1.docx]

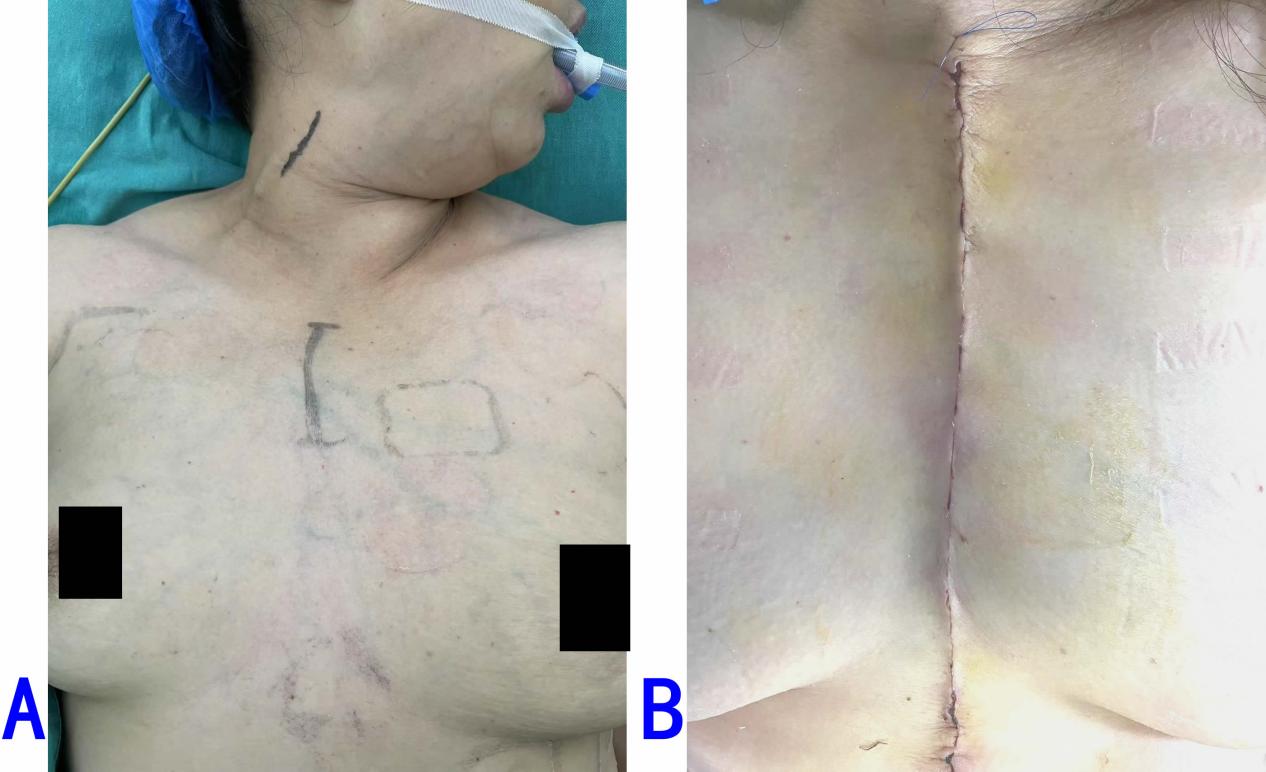
**Supplementary Figure S1.**Systemic venous congestion before and after surgery.(A)Preoperative supine view showing distended external jugular vein and prominent subcutaneous collateral veins on the chest and abdominal walls. (B) Postoperative view showing resolved chest wall venous collaterals.


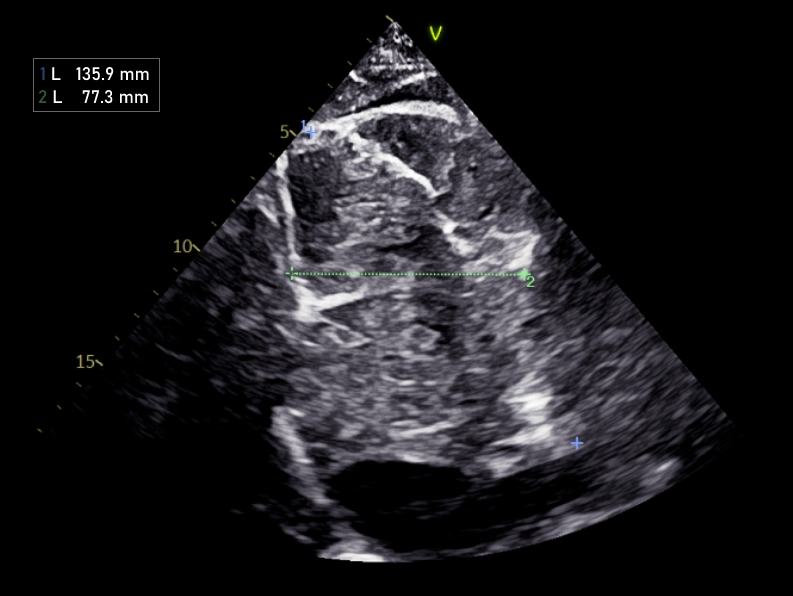


**Supplementary Figure S2.** Subxiphoid cardiac ultrasound view showing the heterogeneous aneurysm adjacent to the right atrial wall.


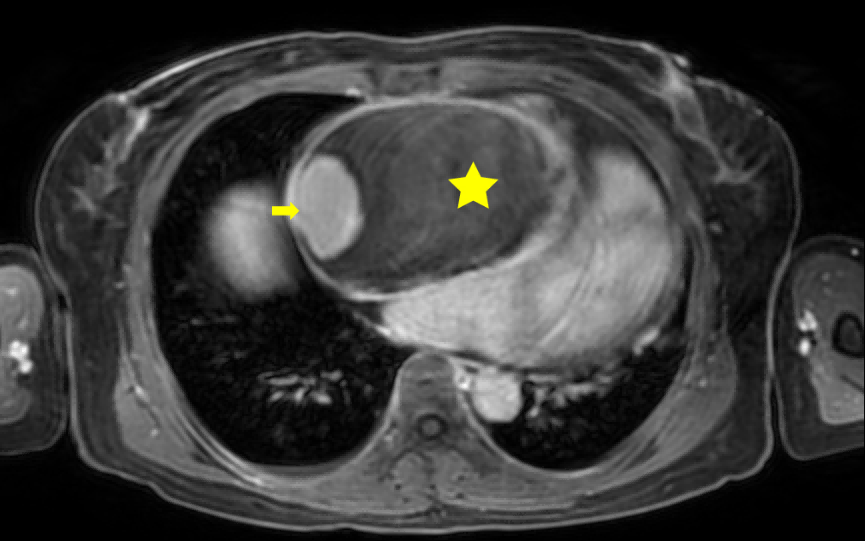


**Supplementary Figure S3.** Cardiac contrast-enhanced MRI displaying the aneurysm; *Arrows indicate blood flow within the lumen; five-pointed star* denotes thrombus.





**Supplementary Figure S4.**  Central venous pressure measurement before and after surgery.(A) Preoperative central venous pressure under anesthesia was 16.7 cm H₂O. (B) Postoperative central venous pressure decreased to 4.3 cm H₂O.


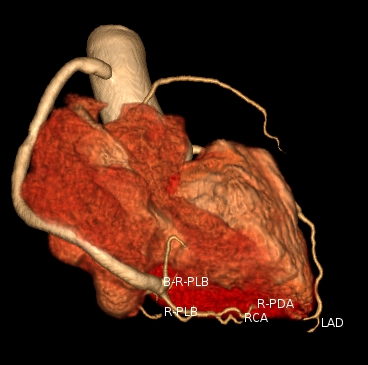


**Supplementary Figure S5** Coronary CTA confirmed a patent lumen in the saphenous vein graft
